# Disease Severity and Risk Factors of 30-Day Hospital Readmission in Pediatric Hospitalizations for Pneumonia

**DOI:** 10.3390/jcm11051185

**Published:** 2022-02-23

**Authors:** Motomori O. Lewis, Phuong T. Tran, Yushi Huang, Raj A. Desai, Yun Shen, Joshua D. Brown

**Affiliations:** 1 Center for Drug Evaluation & Safety, Department of Pharmaceutical Outcomes and Policy, University of Florida College of Pharmacy, Gainesville, FL 32610, USA; motomorilewis@ufl.edu (M.O.L.); phuong.tran@ufl.edu (P.T.T.); yushi.h@ufl.edu (Y.H.); raj.desai@ufl.edu (R.A.D.); yunshen@ufl.edu (Y.S.); 2 Faculty of Pharmacy, Ho Chi Minh City University of Technology (HUTECH), Ho Chi Minh City 700000, Vietnam

**Keywords:** pediatric pneumonia, hospital readmission, healthcare quality, hospital costs

## Abstract

Pneumonia is the leading cause of hospitalization in pediatric patients. Disease severity greatly influences pneumonia progression and adverse health outcomes such as hospital readmission. Hospital readmissions have become a measure of healthcare quality to reduce excess expenditures. The aim of this study was to examine 30-day all-cause readmission rates and evaluate the association between pneumonia severity and readmission among pediatric pneumonia hospitalizations. Using 2018 Nationwide Readmissions Database (NRD), we conducted a cross-sectional study of pediatric hospitalizations for pneumonia. Pneumonia severity was defined by the presence of respiratory failure, sepsis, mechanical ventilation, dependence on long-term supplemental oxygen, and/or respiratory intubation. Outcomes of interest were 30-day all-cause readmission, length of stay, and cost. The rate of 30-day readmission for the total sample was 5.9%, 4.7% for non-severe pneumonia, and 8.7% for severe pneumonia (*p* < 0.01). Among those who were readmitted, hospitalizations for severe pneumonia had a longer length of stay (6.5 vs. 5.4 days, *p* < 0.01) and higher daily cost (USD 3246 vs. USD 2679, *p* < 0.01) than admissions for non-severe pneumonia. Factors associated with 30-day readmission were pneumonia severity, immunosuppressive conditions, length of stay, and hospital case volume. To reduce potentially preventable readmissions, clinical interventions to improve the disease course and hospital system interventions are necessary.

## 1. Introduction

Approximately 8–30% of all pediatric hospital admissions are potentially preventable [1,2,3], costing the healthcare system up to USD 561.6 million in 2017 [1]. Since the passage of the Patient Protection and Affordable Care Act, hospital readmissions have become a measure of healthcare quality promoted by insurers and policymakers to reduce excess healthcare expenditures [4,5,6]. Hospitals failing to meet readmission benchmarks may be subject to reduced reimbursement from the Centers for Medicaid & Medicare Services (CMS) [5,6]. For children’s hospitals, which were exempt from reimbursement penalties, readmission remains a quality measure upheld by several state Medicaid agencies [2]. Based on the influence of these organizations, many children’s hospitals have implemented practices to reduce readmissions and improve hospital care. Readmissions serve as an indicator of inadequate disease management during an initial visit. They also reflect the comprehensiveness of inpatient care including discharge processes, patient education, and follow-up care [7].

Amid national efforts to reduce hospital readmissions and unnecessary healthcare utilization, pneumonia is the single leading cause of hospitalization in pediatric patients [8]. Globally, pneumonia is among the top causes of death in children under 5 years [9]. In addition to its high morbidity and mortality, the economic burden of inpatient care for pneumonia is substantial at nearly USD 6.5 billion [10]. Pneumonia can present with a host of complications. Factors that influence the severity of pneumonia include age, malnutrition, underlying chronic conditions, and timeliness and appropriateness of treatment [11,12,13]. Treatment of pneumonia is hindered by a lack of established criteria to define severity and standardized care plans that address complications [11]. Most children’s hospitals rely on guideline recommendations to generate institution-specific process of care plans for complicated pneumonia. Disease severity plays a large role in pneumonia progression, especially regarding adverse health outcomes.

We hypothesize that the rate of hospital readmissions will be higher in patients with severe pneumonia. To our best knowledge, no prior studies have evaluated the association between disease severity and 30-day all-cause readmission in pediatric pneumonia patients. Additionally, despite many policies, hospital readmission rates did not improve from 2010–2016 [14], yet infection diagnostics, vaccine promotion, and medical care have advanced in recent years [15,16,17,18]. Therefore, the aim of this study was to examine pneumonia readmission rates by severity and evaluate the association between pneumonia severity and 30-day all-cause readmission in pediatric pneumonia hospitalizations using the 2018 Nationwide Readmissions Database (NRD).

## 2. Materials and Methods

We conducted a cross-sectional analysis using hospital discharge data from the 2018 Nationwide Readmissions Database (NRD) curated by Healthcare Cost and Utilization Project (HCUP), Agency for Healthcare Research and Quality (AHRQ). NRD is an all-payer database detailing inpatient admission records from U.S. community hospitals [19]. It includes about 17 million unweighted and 36 million weighted hospitalizations per year. Hospitalizations for any condition among all ages are included in the sample. NRD is a multi-level dataset in that it includes both discharge-level and hospital-level data. To facilitate national readmissions estimates and account for over- or under-sampling, discharge weights are applied through post-stratification on patient and hospital characteristics. Further, NRD allows tracking of individual patients with a unique patient linkage variable that serves as a patient identifier across hospitalizations within one state throughout a calendar year. As a result, individuals seeking care at different hospitals may be identified. The NRD sampling frame covers 27 geographically distributed states across the nation (a full list of HCUP data partners can be found here: https://www.hcup-us.ahrq.gov/db/hcupdatapartners.jsp; accessed 31 January 2022), representing the majority of all U.S. hospitalizations (56.6%) and residents (57.8%). NRD is part of the HCUP family of databases curated for healthcare researchers; the project is supported by the Agency for Healthcare Research and Quality. All databases within the collection are publicly available for purchase.

**Figure 1:**
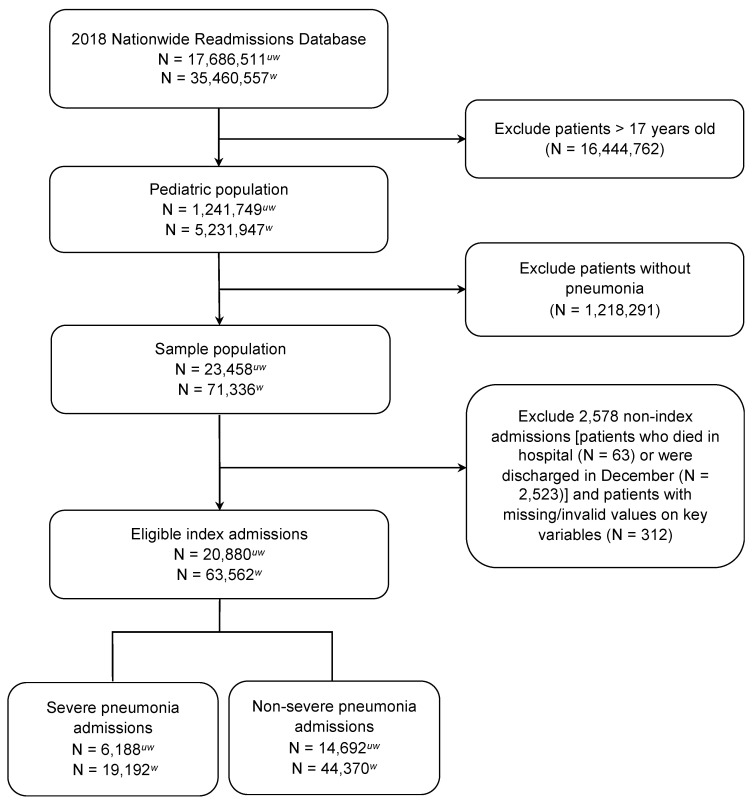
Flow diagram for study sample selection. uw = unweighted; w = weighted.

Our study sample consists of hospitalizations in a pediatric cohort under 18 years of age. To identify pneumonia cases, we selected all hospitalizations with a primary International Classification of Diseases, Tenth Revision, Clinical Modification (ICD-10-CM) diagnosis code for pneumonia (ICD codes shown in Supplement Table S1). To further identify eligible cases, we also selected cases with a primary diagnosis for either septicemia (038.xx) or acute respiratory failure (518.8x) and secondary diagnosis for pneumonia because these conditions are often related to pneumonia. Our definition of pneumonia is developed from an administrative data-based algorithm by Whittle et al., which was shown to have 84% sensitivity, 86% specificity, and 92% PPV [20]. Similar algorithms have been employed in previous studies [21,22] and validation confirmed that using a claims-based algorithm yields similar performance to definite pneumonia cases confirmed by laboratory and radiographic evidence [23]. Patients with missingness on key variables (length of stay, total charge, death, visit link, and days to event) are excluded. To qualify as an index admission eligible for readmissions analysis, patients must not have died in hospital during their visit and must have been discharged from the hospital before 1 December 2015, to provide a 30-day time frame for analyzing readmissions. In our readmissions sample, we excluded patients with an elective hospital admission. The sample selection flow diagram is shown in Figure 1.

After identifying qualifying hospitalizations, the sample was then stratified into severe and non-severe pneumonia cases. To assess pneumonia severity, we constructed a unique severity indicator developed from patient comorbidities and hospital procedures. Based on the Pediatric Infectious Diseases Society–Infectious Diseases Society of America and British Thoracic Society clinical guidelines, we considered severe admissions to be those that included any of the following conditions: respiratory failure, sepsis, mechanical ventilation, dependence on long-term supplemental oxygen, and/or respiratory intubation [24,25]. We define the ICD-9/10-CM/PCS diagnostic and procedural codes for this definition in Table S1. All other pneumonia admissions were considered non-severe. Patient-level (clinical and demographic) and hospital-level characteristics were selected as potential risk factors for readmission. Using the HCUP Elixhauser Comorbidity Software [26], we calculated the Elixhauser mortality risk score, which is an index of comorbidity measures generated from diagnosis codes on the patient record [27]. We also added patient demographics (e.g., age, gender, income quartile, patient location, and state residency), relevant clinical comorbidities (e.g., asthma, acute bronchitis/bronchiolitis, cancer, chronic pulmonary disease, sickle cell disease, and cystic fibrosis; Table S1), and hospital information (e.g., teaching status, bed size, and urban-rural designation). In order to account for the variation in pneumonia cases at each hospital, hospital case volume was calculated by totaling the number of pneumonia cases for each hospital [28].

Our primary outcome of interest was 30-day all-cause hospital readmission rate. To examine the clinical and economic burden, we determined the length of stay, and daily and total hospitalization cost. Cost was calculated as the product of total charge and the hospital-specific cost-to-charge ratio provided by HCUP [29].

Descriptive and summary statistics were reported among the total sample and by pneumonia severity. We compared patient and hospitalization characteristics across the two groups to examine significant differences. For continuous variables, we conducted independent t-tests. For binary and multi-level categorial variables, we used Chi-Square and Fisher’s exact tests, respectively. Associations between pneumonia severity and readmission were evaluated using logistic regression to estimate the odds ratio with 95% confidence intervals. Covariates that differed significantly across severity groups and identified as potential confounders were adjusted for in the model. To ensure the robustness of our findings, we performed additional sensitivity analyses: (1) we adjusted the outcome to include only pneumonia-specific readmissions and (2) we used a second measure of severity—the HCUP severity variable included in the NRD dataset. We applied an a priori significance level of α = 0.05. All analyses were conducted using SAS software 9.4, SAS Institute Inc., Cary, NC, USA.

## 3. Results

Our nationally representative study sample included a total of 20,880 hospitalizations for pediatric pneumonia (Figure 1). Of the sample, one-third (29.6%) were classified as admissions for severe pneumonia (Table 1). In the overall cohort, nearly 90% of hospitalized patients were age 12 years and under. Females comprised 47% of the study sample. The greatest proportion of patients resided in areas with the lowest income quartile (34.8%) and large central metropolitan counties. Three-fourths (74.5%) of admissions originated in the emergency department and 25.0% were admitted on a weekend. Majority of patients had an expected primary payer of Medicaid (60.2%) followed by private insurance (34.5%). Overall, we found a high proportion of respiratory comorbidities (asthma (34.7%), acute bronchitis/bronchiolitis (13.4%), and chronic pulmonary disease (35.4%)). The prevalence of immunosuppressive conditions was low in our sample (cancer (0.9%), cystic fibrosis (2.2%), and sickle cell disease (1.3%)). Most hospitalizations for pneumonia occurred in large (63.9%), teaching hospitals (80.4%) in metropolitan areas (89.0%).

There were significant differences in the clinical and hospital characteristics between non-severe and severe pneumonia admissions. The prevalence of respiratory conditions was significantly higher in the severe pneumonia group than the non-severe group [asthma (36.3 vs. 34.0%, *p* < 0.01), acute bronchitis/bronchiolitis (19.8 vs. 10.8%, *p* < 0.01), and chronic pulmonary disease (37.0 vs. 34.8%, *p* < 0.01)]. Apart from cancer (0.9% for both groups, *p* = 0.70), the frequency of admissions with immunosuppressive conditions was significantly lower among those with severe pneumonia compared to non-severe pneumonia (cystic fibrosis (0.5 vs. 3.0%, *p* < 0.01), and sickle cell disease (0.4 vs. 1.8%, *p* < 0.01). Hospitals within the 76th–100th percentile of pneumonia case volume admitted a greater proportion of severe pneumonia cases than non-severe cases (35.2 vs. 20.7%, *p* < 0.01).

### 3.1. Readmissions and Related Clinical and Economic Burden

The rate of 30-day all-cause readmissions was 5.9% for the total sample, 4.7% for non-severe pneumonia and 8.7% for severe pneumonia (*p* < 0.01; Table 2). Of the total readmissions, 28.4% were specifically for pneumonia. Compared to the index admission, hospital stays for readmissions were generally longer and cost more per day. Among those who were readmitted, hospitalizations for severe pneumonia had a longer length of stay (6.5 vs. 5.4 days, *p* < 0.01) and higher daily cost (USD 3246 vs. USD 2679, *p* < 0.01) than admissions for non-severe pneumonia. The total economic burden for pediatric pneumonia hospital admissions costs over USD 205 million, with severe pneumonia admissions resulting in the majority of costs (>USD 111 million). Thirty-day readmissions for pneumonia contributed to an excess of USD 22 million for hospitalization costs in 2018.

Eight of the 10 most common reasons for readmission were respiratory-related conditions such as pneumonia (23.8%), respiratory failure, insufficiency, or arrest (11.8%), acute bronchitis (7.7%), asthma (6.8%), influenza (3.9%), upper respiratory infections (2.9%), aspiration pneumonitis (2.7%) and respiratory signs and symptoms (e.g., epistaxis, hemorrhage, cough, dyspnea, shortness of breath; 2.5%). Other reasons for readmission included epilepsy or convulsions (3.6%) and septicemia (3.5%) (Table 3).

### 3.2. Risk Factors for Readmission

Hospital admissions for severe pneumonia were associated with 44% increased odds of all-cause 30-day readmission compared to non-severe pneumonia admissions (OR 1.44, 95% CI 1.26–1.65) (Table 4). Higher odds of readmission were seen among children insured through Medicare (OR 2.17, 95% CI 1.20–3.95) or Medicaid (1.20, 95% CI 1.05–1.37). Patients with comorbidities such as cancer (OR 4.82, 95% CI 3.43–6.77) or sickle cell disease (OR 2.14, 95% CI 1.45–3.16) and admissions at metropolitan teaching hospitals (OR 1.48, 95% CI 1.12–1.97) or hospitals with a higher pneumonia case volume (26th–50th percentile: OR 1.33, 95% CI 1.08–1.64; 51st–75th percentile: OR 1.40, 95% CI 1.13–1.74; 76th–100th percentile: OR 1.50, 95% CI 1.22–1.86) were also at increased odds of readmission. Admissions with an increased length of stay (OR 1.04, 95% CI 1.03–1.06) and daily cost (51st–75th percentile: OR 1.29, 95% CI 1.07–1.55; 76th–100th percentile: OR 1.40, 95% CI 1.16–1.69) were indicative of greater odds of readmission. Those less likely to be readmitted were children aged 1–4 years (OR 0.81, 95% CI 0.67–0.98), self-insured patients (OR 0.44, 95% CI 0.22–0.86), and patients diagnosed with asthma (OR 0.78, 95% CI 0.68–0.90).

Both sensitivity analyses were consistent with our main analysis (Tables S2 and S3). When restricting the outcome to pneumonia-specific readmissions, only pneumonia severity (OR 1.48, 95% CI 1.16–1.86), female sex (OR 1.27, 95% CI 1.03–1.58), and hospitals with higher case volume were statistically significant for increased odds of readmission (51st–75th percentile: OR 1.61, 95% CI 1.09–2.38; 76–00th percentile: OR 2.03, 95% CI 1.38–2.99). Applying the HCUP severity class resulted in greater odds of readmission among severe pneumonia cases than our primary severity definition (moderate loss of function: OR 1.76, 95% CI 1.43–2.15; major loss of function: OR 3.93, 95% CI 3.19–4.85; extreme loss of function: OR 5.53, 95% CI 4.35–7.04), and significantly reduced odds of readmission among patients with cystic fibrosis (OR 0.66, 95% CI 0.47–0.93).

## 4. Discussion

Our nationally representative, cross-sectional study is among the first to demonstrate that pneumonia severity is associated with 30-day all-cause readmission in pediatric pneumonia hospitalizations. Patient demographics, such as being publicly insured, was a non-modifiable risk factor for readmission while age 1–4 years and self-pay were protective factors. Patients with immunosuppressive conditions such as cancer and sickle cell disease were more likely to be readmitted than those with other clinical comorbidities, despite these diagnoses being rare and more prevalent among non-severe pneumonia cases. In addition, longer length of stay during an index hospitalization contributed to greater risk of readmission. Finally, we found that hospital case volume and teaching status were more predictive of readmission risk than hospital size.

Our sample had a 30-day all-cause readmission rate of 5.9%. These results corroborate previous literature on the sparse (<10%) readmission prevalence for pediatric pneumonia [30,31,32]. Hospital readmission was more likely among patients with severe pneumonia. Despite conflicting evidence on whether disease severity is associated with readmission [33], there is a need to account for severity in future prediction models for pneumonia readmission. Risk stratification is a useful method to identify patients with more complications for treatment interventions and reduce the likelihood of rehospitalization [34]. Special attention is needed for children with immunosuppressive conditions. It is well established that readmission rates for pediatric pneumonia are higher among patients with chronic conditions [35,36,37,38]. However, in our sample, the presence of immunosuppressive diseases, such as cancer and SCD, showed an increased risk of readmission, while chronic respiratory conditions such as asthma did not. While readmission in SCD and cancer may be attributable to other factors due to their underlying disease, a pneumonia diagnosis presents an added layer of complexity and can lead to prolonged illness and complications. Vaccination can be considered to reduce pneumonia in this high-risk subgroup [37].

Our analysis demonstrated an association between longer length of stay and risk of readmission, as supported by a similar study of children with complicated pneumonia [39]. Contrary to our findings, other observational studies of pediatric readmissions in U.S. children’s hospitals found no meaningful association between index hospitalization length of stay and risk of readmission in children’s hospitals [40,41]. Additionally, increasing length of stay to reduce readmission was not a practical, cost-effective solution for most diagnoses [40]. While there is still conflict on whether index admission length of stay is a valuable measure for predicting readmissions, length of stay may be useful as an indicator of hospital efficiency and quality-of-care. Guideline-concordant therapy has been associated with shorter length of stay in adults hospitalized with community-acquired pneumonia [42]. Future research is needed to explore whether a guideline-directed treatment approach can be extended to the pediatric population and reduce resource utilization for pneumonia. Regarding cost from the payer perspective, patients who were self-insured had significantly lower odds of readmission. This signifies that hospital readmissions are not only influenced by clinical need or the quality of healthcare delivery, but by patients’ ability to pay.

Several hospital-level factors were significant predictors of all-cause readmission. In particular, admissions occurring at hospitals with higher pneumonia case volume and metropolitan teaching status were more likely to result in readmission. Non-modifiable hospital factors such case-mix and location in high-poverty areas have been related to readmission [43]. Metropolitan teaching hospitals, which are typically large, specialized care centers treating complex cases, are more likely affected by these factors. Previous literature found hospital-level factors contributed to nearly 80% of preventable readmissions [44]. Given the significance of hospital variation in readmissions, a systems-based approach may be necessary to prevent readmissions and improve hospital quality. Evidence suggests that discharge transition plans, follow-up care, and addressing adverse social determinants of health are useful interventions to reduce hospital readmission rates [45,46].

Our study has noteworthy strengths. We used a nationally representative sample of admissions from community hospitals within the U.S., providing generalizability to the entire U.S. pediatric population. Further, NRD data is curated from statewide partnerships, permitting the linkage of patients hospitalized in more than one institution within a single state. However, there are also a few limitations to our investigation. Although NRD includes admissions from all hospital units, we were not able to identify admissions that were transferred to the intensive care unit (ICU) due to the lack of availability of UB-04 revenue codes in our data source. Patients with severe pneumonia are often referred for admission in the ICU [25]. Second, our data does not include other relevant clinical information such as respiratory laboratory values, interventions, and medication use. Third, while we were able to track the order of patients’ hospitalizations within the year, our cross-sectional study design is not suitable to determine the timing of events within a hospitalization. Fourth, given that our data source is exclusive to US hospitals, our analysis may not be generalizable to other countries with different pay structures and healthcare delivery systems. Despite varying global economic and healthcare systems, the clinical and demographic risk factors identified in our study may still be applicable to international patient populations.

## 5. Conclusions

Our study examined the clinical and economic burden of pediatric pneumonia readmissions. Hospitalizations for pediatric pneumonia promote a significant economic burden, especially for severe pneumonia. We found that pneumonia severity is associated with risk of readmission. Risk factors for 30-day all-cause readmission also include immunosuppressive conditions, index admission length of stay, and hospital case volume. Both clinical and hospital systems-level interventions are necessary to reduce potentially preventable readmissions.

## Figures and Tables

**Table 1:** Patient and hospital characteristics of pediatric pneumonia admissions, 2018.

Characteristics	Total Sample N = 20,880 (100%)	Non-Severe Pneumonia N = 14,692 (70.4%)	Severe Pneumonia N = 6188 (29.6%)	*p*-Value
**Patient Demographic Characteristics**				
**Age group**				**<0.01**
<1 year	2450 (11.7)	1622 (11.0)	828 (13.4)	
1–4 years	9782 (46.9)	6807 (46.3)	2975 (48.1)	
5–12 years	6504 (31.2)	4747 (32.3)	1757 (28.4)	
13–17 years	2144 (10.3)	1516 (10.3)	628 (10.2)	
**Female**	9882 (47.3)	6956 (47.4)	2926 (47.3)	0.94
**Income quartile**				**<0.01**
0–25th percentile	7271 (34.8)	5293 (36.0)	1978 (32.0)	
26th–50th percentile (median)	5955 (28.5)	4090 (27.8)	1865 (30.1)	
51st–75th percentile	4678 (22.4)	3267 (22.24)	1411 (22.8)	
76th–100th percentile	2976 (14.3)	2042 (13.9)	934 (15.1)	
**Patient location**				**<0.01**
Large central metro	5548 (26.6)	3905 (26.6)	1643 (26.7)	
Large fringe metro	4519 (21.6)	3081 (21.0)	1438 (23.2)	
Medium metro	4373 (20.9)	2964 (20.2)	1409 (22.8)	
Small metro	2405 (11.5)	1768 (12.0)	637 (10.3)	
Micropolitan	2276 (10.9)	1651 (11.2)	625 (10.1)	
Non-metro or micropolitan	1759 (8.4)	1323 (9.0)	436 (7.1)	
**In-state resident**	19,883 (95.2)	14,058 (95.7)	5825 (94.1)	**<0.01**
**Emergency department**	15,559 (74.5)	10,916 (74.3)	4644 (75.1)	0.25
**Admitted on weekend**	5209 (25.0)	3650 (24.8)	1559 (25.2)	0.59
**Season**				0.19
Winter	6292 (30.1)	4437 (30.2)	1855 (30.0)	
Spring	6238 (29.9)	4337 (29.5)	1901 (30.7)	
Summer	3084 (14.8)	2161 (14.7)	923 (14.9)	
Fall	5266 (25.2)	3757 (25.6)	1509 (24.4)	
**Expected primary payer**				**<0.01**
Medicare	125 (0.6)	82 (0.6)	43 (0.7)	
Medicaid	12,567 (60.2)	8728 (59.4)	3839 (62.0)	
Private insurance	7193 (34.5)	5141 (35.0)	2052 (33.2)	
Self-pay	440 (2.1)	336 (2.3)	104 (1.7)	
No charge	555 (2.7)	405 (2.8)	150 (2.4)	
**Patient Clinical Characteristics**				
**Respiratory conditions**				
Asthma	7244 (34.7)	5001 (34.0)	2243 (36.3)	<0.01
Acute bronchitis/bronchiolitis	2805 (13.4)	1579 (10.8)	1226 (19.8)	<0.01
Chronic pulmonary disease	7399 (35.4)	5111 (34.8)	2288 (37.0)	<0.01
**Immunosuppressive conditions**				
Cancer, any type	187 (0.9)	134 (0.9)	53 (0.9)	0.70
Cystic fibrosis	466 (2.2)	434 (3.0)	32 (0.5)	**<0.01**
Sickle cell disease	280 (1.3)	257 (1.8)	23 (0.4)	**<0.01**
**HCUP severity class**				**<0.01**
Minor	6199 (29.7)	5556 (37.8)	643 (10.4)	
Moderate	8199 (39.3)	6754 (46.0)	1445 (23.4)	
Major	4177 (20.0)	1860 (12.7)	2317 (37.4)	
Extreme	2305 (11.0)	522 (3.6)	1783 (28.8)	
**Hospital Characteristics**				
**Hospital size**				**<0.01**
Small	3142 (15.1)	2318 (15.8)	824 (13.3)	
Medium	4406 (21.1)	3413 (23.2)	993 (16.1)	
Large	13,332 (63.9)	8961 (61.0)	4371 (70.6)	
**Hospital urban-rural** **designation**				**<0.01**
Large metropolitan	11,234 (53.8)	7636 (52.0)	3598 (58.1)	
Small metropolitan	7352 (35.2)	5153 (35.1)	2199 (35.5)	
Micropolitan	1609 (7.7)	1293 (8.8)	316 (5.1)	
Non-urban	685 (3.3)	610 (4.2)	75 (1.2)	
**Hospital teaching status**				**<0.01**
Metro non-teaching	1790 (8.6)	1465 (10.0)	325 (5.3)	
Metro teaching	16,796 (80.4)	11,324 (77.0)	5472 (88.4)	
Non-metro hospital	22.94 (11.0)	1903 (13.0)	391 (6.3)	
**Hospital case volume**				**<0.01**
0–25th percentile	5347 (25.6)	4341 (29.5)	1006 (16.3)	
26th–50th percentile (median)	5154 (24.7)	3750 (25.5)	1404 (22.7)	
51st–75th percentile	5165 (24.7)	3567 (24.3)	1598 (25.8)	
76th–100th percentile	5214 (25.0)	3034 (20.7)	2180 (35.2)	

**Table 2:** Clinical and economic burden of pediatric pneumonia readmissions, 2018.

Outcome	Total Sample N = 20,880 (100%)	Non-Severe Pneumonia N = 14,692 (70.4%)	Severe Pneumonia N = 6188 (29.6%)	*p*-Value
30-day readmission, all-cause	1225 (5.9)	687 (4.7)	538 (8.7)	**<0.01**
30-day readmission, pneumonia-specific	348 (1.7)	210 (1.4)	138 (2.2)	**<0.01**
**Index hospitalization**				
Length of stay (days), mean ± SD	3.8 (4.1)	3.0 (2.9)	5.7 (5.7)	**<0.01**
Daily cost (USD), mean ± SD	2341 (1823)	2120 (1363)	2865 (2533)	**<0.01**
Sum of total costs (USD)	205,400,992	93,869,832	111,531,160	**<0.01**
**30-day readmission**				
Length of stay (days), mean ± SD	5.9 (5.9)	5.4 (5.5)	6.5 (6.4)	**<0.01**
Daily cost (USD), mean ± SD	2928 (1963)	2679 (1978)	3246 (1898)	**<0.01**
Sum of total costs (USD)	22,036,400	10,340,098	11,696,302	**<0.01**

**Table 3:** Top 10 causes of readmissions based on Clinical Classifications Software [26] Refined.

Principal Diagnosis	N (%)
Pneumonia (except that caused by tuberculosis)	291 (23.8)
Respiratory failure, insufficiency, or arrest	144 (11.8)
Acute bronchitis	94 (7.7)
Asthma	83 (6.8)
Influenza	48 (3.9)
Epilepsy or convulsions	44 (3.6)
Septicemia	43 (3.5)
Other specified upper respiratory infections	36 (2.9)
Aspiration pneumonitis	33 (2.7)
Respiratory signs and symptoms (e.g., epistaxis, hemorrhage, cough, dyspnea, shortness of breath, etc.)	30 (2.5)

**Table 4:** Risk factors associated with 30-day all-cause readmission.

Covariate	Adjusted Odds Ratio (95% CI)
**Severe pneumonia**	1.44 (1.26, 1.65) *
**Age group**	
<1 year	Reference
1–4 years	0.81 (0.67, 0.98) *
5–12 years	0.88 (0.72, 1.08)
13–17 years	1.25 (0.99, 1.58)
**Female**	0.99 (0.88, 1.12)
**Expected primary payer**	
Private insurance	Reference
Medicare	2.17 (1.20, 3.95) *
Medicaid	1.20 (1.05, 1.37) *
Self-pay	0.44 (0.22, 0.86) *
No charge	1.40 (0.98, 1.98)
**Length of stay**	1.04 (1.03, 1.06) *
**Daily cost**	
0–25th percentile	Reference
26th–50th percentile (median)	1.15 (0.95, 1.38)
51st–75th percentile	1.29 (1.07, 1.55) *
76th–100th percentile	1.40 (1.16, 1.69) *
**Asthma**	0.78 (0.68, 0.90) *
**Cancer, any type**	4.82 (3.43, 6.77) *
**Cystic fibrosis**	0.89 (0.62, 1.27)
**Sickle cell disease**	2.14 (1.45, 3.16) *
**Hospital size**	
Small	Reference
Medium	1.00 (0.81, 1.24)
Large	1.13 (0.95, 1.35)
**Hospital teaching status**	
Non-metropolitan hospital	Reference
Metropolitan non-teaching	0.84 (0.57, 1.23)
Metropolitan teaching	1.48 (1.12, 1.97) *
**Hospital case volume**	
0–25th percentile	Reference
26th–50th percentile (median)	1.33 (1.08, 1.64) *
51st–75th percentile	1.40 (1.13, 1.74) *
76th–100th percentile	1.50 (1.22, 1.86) *

* Odds ratio is statistically significant.

## Data Availability

Data are used under a Data Use Agreement that does not allow distribution of source data. HCUP’s Nationwide and State-Specific Databases are available for purchase. Programming codes are available upon request.

## Supplementary Materials

The following supporting information can be downloaded at: https://www.mdpi.com/article/10.3390/jcm11051185/s1, Table S1: ICD-10 diagnosis and procedure codes for pneumonia and relevant comorbidities; Table S2: Sensitivity analysis 1—Risk factors associated with 30-day pneumonia-specific readmission for pediatric pneumonia; Table S3: Sensitivity analysis 2—Risk factors associated with 30-day all-cause readmission for pediatric pneumonia using HCUP severity class.
