# Supplementary material for: Disease Severity and Risk Factors of 30-Day Hospital Readmission in Pediatric Hospitalizations for Pneumonia
Source: J Clin Med. 2022 Feb 23;11(5):1185. doi: 10.3390/jcm11051185 (PMC8911283; doi:10.3390/jcm11051185)
Supplement: Supplementary file 1 [file jcm-11-01185-s001.zip › jcm-1577369-supplementary.pdf]

Supplemental Materials

Table S1. ICD-10 diagnosis and procedure codes for pneumonia and relevant comorbidities

Diagnosis/Procedure		ICD-10 code type	ICD-10-CM/ICD-10-PCS codes
Pneumonia		ICD-10-CM	J09X1, J100, J1000, J1001, J1008, J110, J1100, J1108, J120, J121, J122, J123, J1281, J1282, J1289, J129, J13, J14, J15, J16, J17, J18, A221, A3701, A3711, A3781, A3791, A481, B250, B440, B7781
Severe pneumonia	Respiratory failure	ICD-10-CM	Newborn: P220, P285, P2881<br>Pediatric: J96, J80, R0603
	Sepsis	ICD-10-CM	A40, A41, R651, R6511, R6520, R6521
	Mechanical ventilation	ICD-10-PCS	5A09357, 5A09457, 5A09557, 5A1935Z, 5A1945Z, 5A1955Z
	Dependence on supplemental oxygen, long-term	ICD-10-CM	Z9981
	Respiratory intubation	ICD-10-CM	09HN7BZ, 09HN8BZ, 0BH13EZ, 0BH17EZ, 0BH18EZ, 0CHY7BZ, 0CHY8BZ, 0DH57BZ, 0DH58BZ, 0WHQ73Z, 0WHQ7YZ
Asthma		ICD-10-CM	J45
Acute bronchitis/bronchiolitis		ICD-10-CM	J20, J21
Cancer (any type)		ICD-10-CM	C00-C96
Chronic pulmonary disease		ICD-10-CM	J410, J411, J418, J42, J430, J431, J432, J438, J439, J440, J441, J449, J4520, J4521, J4522, J4530, J4531, J4532, J4540, J4541, J4542, J4550, J4551, J4552, J45901, J45902, J45909, J45990, J45991, J45998, J470, J471, J479, J60, J61, J620, J628, J630, J631, J632, J633, J634, J635, J636, J64, J65, J660, J661, J662, J668, J670, J671, J672, J673, J674, J675, J676, J677, J678, J679, J684, J701, J703
Cystic fibrosis		ICD-10-CM	E84
Sickle cell disease (SCD)		ICD-10-CM	D5700, D5701, D5702, D5703, D5709, D571, D5720, D57211, D57212, D57213, D57218, D57219, D5740, D57411, D57412, D57413, D57418, D57419, D5742, D57431, D57432, D57433, D57438, D57439, D5744, D57451, D57452, D57453, D57458, D57459, D5780, D57811, D57812, D57813, D57818, D57819

Table S2. Sensitivity analysis 1 – Risk factors associated with 30-day pneumonia-specific readmission for pediatric pneumonia

Covariate	Adjusted Odds Ratio (95% CI)
**Severe pneumonia**	1.48 (1.16, 1.86)*
**Age group**	
< 1 year	Reference
1-4 years	0.87 (0.61, 1.22)
5-12 years	0.95 (0.66, 1.38)
13-17 years	1.23 (0.80, 1.90)
**Female**	1.27 (1.03, 1.58)*
**Expected primary payer**	
Private insurance	Reference
Medicare	0.55 (0.08, 3.99)
Medicaid	1.15 (0.91, 1.45)
Self-pay	1.64 (0.24, 1.76)
No charge	1.50 (0.82, 2.75)
**Length of stay**	1.01 (0.99, 1.04)
**Daily cost**	
0-25th percentile	Reference
26th-50th percentile (median)	0.90 (0.65, 1.23)
51st-75th percentile	0.99 (0.72, 1.35)
76th-100th percentile	0.83 (0.59, 1.15)
**Asthma**	0.83 (0.66, 1.06)
**Cancer, any type**	1.40 (0.57, 3.48)
**Cystic fibrosis**	1.50 (0.82, 2.73)
**Sickle cell disease**	1.68 (0.78, 3.61)
**Hospital size**	
Small	Reference
Medium	1.26 (0.87, 1.84)
Large	1.00 (0.72, 1.40)
**Hospital teaching status**	
Non-metropolitan hospital	Reference
Metropolitan non-teaching	0.51 (0.26, 1.00)
Metropolitan teaching	0.92 (0.59, 1.44)
**Hospital case volume**	
0-25th percentile	Reference
26th-50th percentile (median)	1.37 (0.94, 2.00)
51st-75th percentile	1.61 (1.09, 2.38)*
76th-100th percentile	2.03 (1.38, 2.99)*

*Odds ratio is statistically significant

Table S3. Sensitivity analysis 2 – Risk factors associated with 30-day all-cause readmission for pediatric pneumonia **using HCUP severity class**

Covariate	Adjusted Odds Ratio (95% CI)
**HCUP severity class**	
Minor loss of function	Reference
Moderate loss of function	1.75 (1.42, 2.14)*
Major loss of function	3.92 (3.18, 4.83)*
Extreme loss of function	5.48 (4.31, 6.98)*
**Age group**	
< 1 year	Reference
1-4 years	0.83 (0.68, 1.00)
5-12 years	0.83 (0.68, 1.02)
13-17 years	1.06 (0.84, 1.33)
**Female**	1.02 (0.91, 1.15)
**Expected primary payer**	
Private insurance	Reference
Medicare	1.89 (1.02, 3.42)*
Medicaid	1.15 (1.01, 1.31)*
Self-pay	0.44 (0.22, 0.86)*
No charge	1.29 (0.90, 1.84)
**Length of stay**	1.01 (1.00, 1.03)
**Daily cost**	
0-25th percentile	Reference
26th-50th percentile (median)	1.04 (0.86, 1.26)
51st-75th percentile	1.09 (0.91, 1.32)
76th-100th percentile	1.06 (0.87, 1.28)
**Asthma**	0.76 (0.66, 0.86)*
**Cancer, any type**	3.93 (2.79, 5.52)*
**Cystic fibrosis**	0.66 (0.47, 0.93)*
**Sickle cell disease**	1.87 (1.26, 2.78)*
**Hospital size**	
Small	Reference
Medium	0.99 (0.80, 1.23)
Large	1.04 (0.87, 1.25)
**Hospital teaching status**	
Non-metropolitan hospital	Reference
Metropolitan non-teaching	0.77 (0.52, 1.14)
Metropolitan teaching	1.20 (0.90, 1.60)
**Hospital case volume**	
0-25th percentile	Reference
26th-50th percentile (median)	1.26 (1.02, 1.55)*
51st-75th percentile	1.26 (1.02, 1.57)*
76th-100th percentile	1.28 (1.03, 1.58)*

*Odds ratio is statistically significant
